# Microstructure and Mechanical Properties of Mg-8Li-3Al-0.3Si Alloy Deformed Through a Combination of Back-Extrusion and Spinning Process

**DOI:** 10.3390/ma18020417

**Published:** 2025-01-17

**Authors:** Changzhen Jia, Kunkun Deng, Cuiju Wang, Kaibo Nie, Quanxin Shi, Yijia Li, Pengcheng Tian

**Affiliations:** Shanxi Key Laboratory of Magnesium-Based Materials, College of Materials Science and Engineering, Taiyuan University of Technology, Taiyuan 030024, China; jiachangzhen0711@163.cm (C.J.); wangcuiju@tyut.edu.cn (C.W.); niekaibo@tyut.edu.cn (K.N.); shiquanxin@tyut.edu.cn (Q.S.); liyijia@tyut.edu.cn (Y.L.); tianpengcheng@163.com (P.T.)

**Keywords:** Mg-Li alloy, spinning, laminar structure, work hardening behavior, softening behavior

## Abstract

In this work, the Mg-8Li-3Al-0.3Si (LAS830) alloy was prepared by the vacuum melting method. The as-cast alloy was subjected to backward extrusion at 250 °C and then spun at 250 °C. The microstructure and mechanical properties of the alloy during deformation were studied. The results showed that the LAS830 alloy primarily consisted of α-Mg and β-Li phases, and the AlLi, MgLi_2_Al, and Mg_2_Si phases were dispersed. After backward extrusion, the grains and AlLi phase were refined, the β-Li phase recrystallized, and the fine MgLi_2_Al phase precipitated. The spinning of the extruded alloy tubes resulted in the lamellar distribution of an α/β duplex microstructure, with even finer grains and more dispersed precipitates. The combined deformation significantly enhanced the alloy’s strength and ductility, with the ultimate tensile strength reaching 235.4 MPa and an elongation of 15.74%. In addition, the average hardness of the α/β phase increases after backward extrusion, but the average hardness of the β-Li phase increases further after spinning. The as-cast LAS830 alloy exhibited a high work hardening rate but a low softening rate. With reverse extrusion, the work hardening rate decreased and the softening degree increased. Compared with backward extrusion, the work hardening rate and softening degree of the LAS830 alloy was reduced after spinning due to the combined effect of the lamellar distributed duplex microstructure and the dispersed second phases in the alloy, while its softening rate increased.

## 1. Introduction

Magnesium–lithium (Mg-Li) alloys are considered promising materials for applications in aerospace, transportation, and electronic information due to their low density, high specific strength and stiffness, and excellent electromagnetic shielding capabilities [1,2,3]. With the increase in lithium (Li) content, significant changes are noted in the microstructure and mechanical properties of Mg-Li alloys. When the Li content is below 5.7 wt.%, Li atoms are solid-solved into the Mg matrix, forming a single-phase α-Mg structure with a hexagonal close-packed (HCP) arrangement, resulting in alloys with high strength but poor ductility. When the Li content surpasses 10.3 wt.%, Mg atoms are solid-solved in lithium metal, forming a single-phase β-Li structure with a body-centered cubic (BCC) arrangement. This formation leads to an enhancement in ductility, albeit at the cost of reduced strength. When the Li content is between 5.7 wt.% and 10.3 wt.%, a dual-phase microstructure of α-Mg + β-Li is exhibited by the alloys, which combined the strength of the α-Mg phase with the ductility of the β-Li phase, achieving an effective balance between the two [4]. However, unlike traditional magnesium alloys, although Mg-Li alloys have good ductility, the absolute strength of binary alloys is relatively low, which limits their widespread application [5,6].

Alloying is one of the primary methods for enhancing the strength of Mg-Li alloys [7]. Aluminum (Al) is one of the most vital alloying elements for Mg-Li alloys, as its density is close to that of Mg and it has a high solubility in Mg, which helps maintain the lightweight characteristic of Mg-Li alloys [8]. Furthermore, the addition of Al can generate AlLi and MgLi_2_Al phases with the matrix, providing precipitation strengthening and significantly improving the alloy’s strength [9,10]. However, an excessive amount of Al can lead to over-aging softening issues, causing instability in the microstructure and properties of the alloy [11]. Researchers addressed these issues by introducing stable secondary phases [12]. Silicon (Si) can form high-melting-point, high-hardness secondary phases with Mg, such as Mg_2_Si, thereby enhancing the thermal stability of the alloy [13]. Additionally, the Mg_2_Si phase can promote the tensile strength of Mg-Li alloys. Shi et al. [14] introduced Si into Mg-Li alloys in the form of an Al-12.5%Si master alloy, and the resulting Mg-8Li-3(Al-Si) alloy showed superior strength compared to the Mg-8Li alloy, with a 100% increase in tensile strength. Therefore, the co-addition of Al and Si is beneficial for the performance improvement of Mg-Li alloys.

Thermal deformation is an effective means to enhance the mechanical properties of alloys and achieve plastic forming [4]. The primary forming techniques for magnesium alloy tubes encompass extrusion, spinning, and stamping processes, among others [15]. Among them, backward extrusion, as a traditional plastic forming technique for tubular parts, is widely used in the preparation of magnesium alloy components [16]. Zhao et al. [17] prepared AZ80 alloy tubes with fine grains and excellent properties through backward extrusion. Spinning forming, as an advanced forming process, has the advantages of low forming load, extended material forming limit, and high material utilization rate compared with other metal forming technologies [18]. Jin et al. [19] found that spinning promoted the dynamic recrystallization of Mg alloys, with significant grain refinement. A mass of second phases precipitate and gradually refine with the progress of spinning. Through spinning, grain refinement and enhancement of mechanical properties were achieved in AZ80 alloy tubes by Cao et al. [20]. In addition, it was shown that combined deformation has an essential effect on grain refinement and the improvement in mechanical properties of magnesium alloys [21]. Huang et al. [22] prepared AZ91 alloy tubes through the combined deformation of backward extrusion and spinning, with grain size refining from 48.3 μm after backward extrusion to 9.6 μm after spinning. It is evident that backward extrusion can be used as a method for preparing spinning billets. However, research on backward extrusion or spinning forming in the field of magnesium alloys mainly focuses on the AZ series, and there is relatively limited research on the microstructure and mechanical properties of Mg-Li alloy spinning forming, and further research is needed on the microstructure evolution and hardening and softening mechanisms of Mg-Li alloys during the combined deformation process.

In this work, the Mg-8Li-3Al-0.3Si (LAS830) alloy was prepared based on the vacuum melting method. Subsequently, the as-cast alloy underwent a combination of backward extrusion and spinning deformation. The effects of the combined deformation on the microstructure and mechanical properties of the alloy were investigated, and the work hardening and softening behavior of the LAS830 alloy were discussed.

## 2. Experimental Procedures

**Figure 1:**
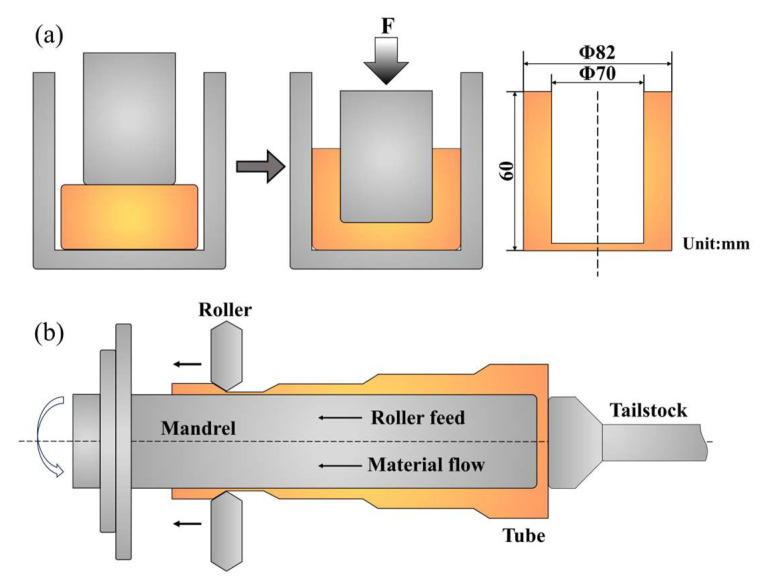
(**a**) Back extrusion diagram and dimensions; (**b**) spinning schematic diagram.

The raw materials for the preparation of Mg-8Li-3Al-0.3Si (LAS830) alloy consist of pure magnesium ingots (99.9 wt.%, Shanxi Yinguang Huasheng Magnesium Industry Co., Ltd, Shanxi, China), pure aluminum ingots (99.9 wt.%, Shanxi Yinguang Huasheng Magnesium Industry Co., Ltd, Shanxi, China), pure lithium granules (99.99 wt.%, Zhongnuo New Materials (Beijing) Technology Co., Ltd, Beijing, China), and Al-27wt.%Si (Jiangsu Haoran Spray Forming Alloy Co., Ltd.) hypereutectic alloy, with the latter being prepared through the spray deposition process. Melting preparation was conducted in a vacuum resistance smelting furnace, with the entire process maintained in an atmosphere of Ar and SF_6_. The alloy ingot melted at 720 °C. It was then stirred with a paddle for 3 min and held for an additional 10 min before being poured into a mold that was preheated to 250 °C, resulting in the acquisition of the alloy ingot. From the ingot, cylindrical blanks of Φ80 × 30 mm were cut, and subsequently, these blanks were backward extruded at 250 °C to form tubes with an inner diameter of 70 mm and a wall thickness of 6 mm, as shown in Figure 1a. The tubes were then heated and spun using a pair of wheel spinning machines, with the schematic diagram shown in Figure 1b. Initially, the tubes were heated to 250 °C and held for 60 min, while the spinning core was preheated to 250 °C. During the spinning process, the core spindle rotated at 240 r/min, and the spinning wheel’s feed rate was 0.1 mm/r. The reduction in the first pass was 20%, and for subsequent passes, it was 10%. After 8 passes of spinning, the final reduction reached 60%, successfully obtaining a LAS830 alloy tube with a wall thickness of 2.5 mm. For the convenience of explanation, the tubes obtained from backward extrusion and spinning are referred to as BE and SP, respectively.

**Figure 2:**
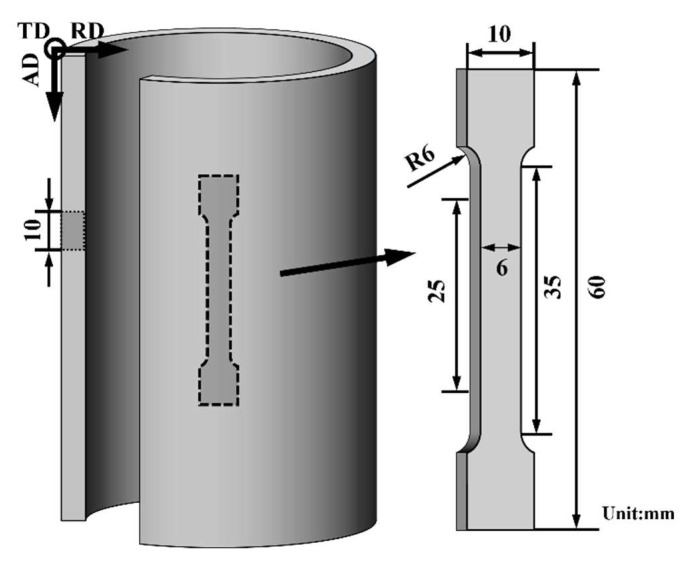
Sampling position and dimensions of the tensile test specimen.

The radial, axial, and tangential directions of the alloy tube are denoted as RD, AD, and TD, respectively. As shown in Figure 2, metallographic samples were sectioned along the RD-AD plane. After mechanical grinding and polishing, the samples were etched using oxalic acid solution (2 g oxalic acid + 100 mL H_2_O). Subsequently, the microstructure of the samples was observed under an optical microscope (OM, OLYMPUS-GX53, OLTMPUS, Tokyo, Japan), scanning electron microscope (SEM, JSM-IT700HR, JEOL, Tokyo, Japan), and electron backscatter diffraction system (EBSD, JEOL JSM-7000 F, JEOL, Tokyo, Japan). The fracture morphology after tensile testing was also observed under the scanning electron microscope, with the preparation method for the side fracture being the same as that for the metallographic samples. The microstructure was further observed using a transmission electron microscope (TEM, JEM-2100F, JEOL, Tokyo, Japan). Phase composition was determined using an X-ray diffractometer (XRD, RIGAKU-SMARTLAB, RIGAKU, Tokyo, Japan) with a scanning angle range of 20–80° and a scanning speed of 5 (°)/min. XRD phase analysis was conducted using MDI Jade 6.0.

Uniaxial tensile tests of as-cast alloys and alloy tubes were carried out on a universal testing machine. The sampling position and sample size of the alloy tube are shown in Figure 2. The tensile specimen of backward extrusion and spinning alloy tube was selected at the position of the tube core. In order to ensure the accuracy of the tensile data, no less than 4 samples were used for each state of the alloy. The tests were performed at room temperature with an electronic universal tensile testing machine (MTS (E45.105)). The tensile direction of the samples was parallel to the spinning direction (AD), and the tensile speed was set at 0.5 mm/min. The sampling position and specimen dimensions are shown in Figure 2. Stress relaxation tests were also conducted on this equipment, with the initial strain value of the first cycle set at 1.2% and each strain gap of 0.5%. The unloading time in each cycle was 600 s. The nanoindentation testing of the alloy was performed using the Hysitron TI 980 nanoindentation instrument (BURKER, Billerica, MA, USA). During the test, a 12 × 12 quadrilateral lattice with a point spacing of 5 μm was tested using a load of 8000 μN.

## 3. Result

### 3.1. Microstructure

**Figure 3:**
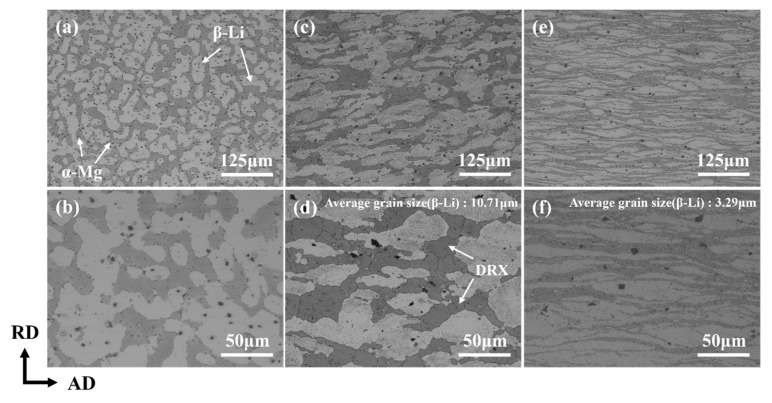
The OM microstructures of LAS830 alloy in as-cast and combination deformation: (**a**,**b**) as-cast; (**c**,**d**) BE; (**e**,**f**) SP.

The OM images of the LAS830 alloy in as-cast and combination deformation are depicted in Figure 3. It was revealed that the LAS830 alloy is characterized by a duplex microstructure. The white bright phase in the as-cast alloy is α-Mg phase, and the β-Li phase is represented by the gray dendrite phase. The OM images of the alloy after backward extrusion deformation, as shown in Figure 3c,d. The dual-phase structure is distributed along the backward extrusion direction, the gray β-Li phase becomes coarse, the grain boundary is bent inward, and equiaxed grains appear. The appearance of equiaxed grains indicates the occurrence of dynamic recrystallization (DRX) in the β-Li phase [23]. The average grain size is about 10.71 μm. After spinning, both the α-Mg and β-Li phases are stretched with the AD, the interlamellar spacing between the α/β phases is reduced, and the average size of grain in β-Li phase is approximately 3.29 μm. This phenomenon is attributed to the concurrent influence of the axial shear stress in the AD and the radial compressive stress in the RD during the spinning process.

**Figure 4:**
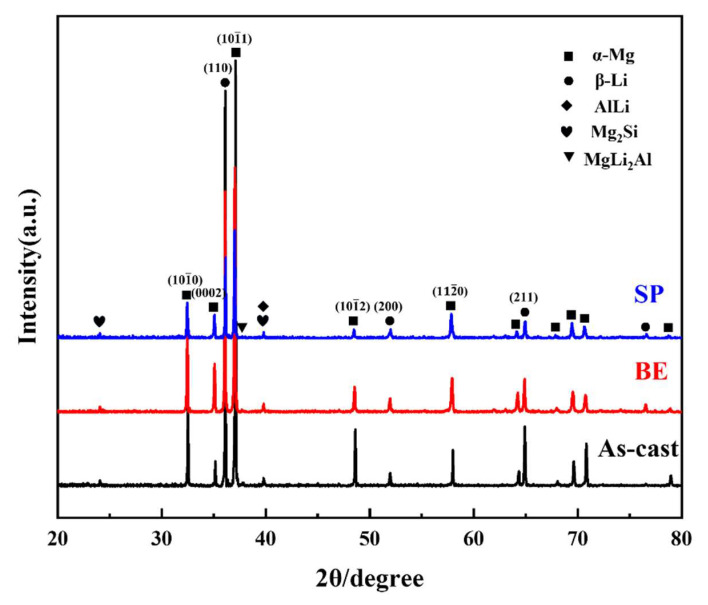
The XRD patterns of LAS830 alloy in as-cast and combination deformation.

To determine the phase composition of the LAS830 alloy in various states, X-ray diffraction (XRD) phase analysis was conducted on the alloy, and the results are shown in Figure 4. The cast LAS830 alloy is mainly composed of α-Mg, β-Li, AlLi, Mg_2_Si, and MgLi_2_Al phases. Thermal deformation does not change the composition of the phase. Due to its thermal stability, the diffraction peak intensity of Mg_2_Si phase does not change significantly [13]. As the degree of deformation increases, the diffraction peak intensity of the α-Mg phase and β-Li phase gradually decreases. The diffraction peak intensity of the MgLi_2_Al phase and AlLi phase also changes, indicating that the grain size or orientation of the matrix and the content of the second phase may change during the composite deformation process.

**Figure 5:**
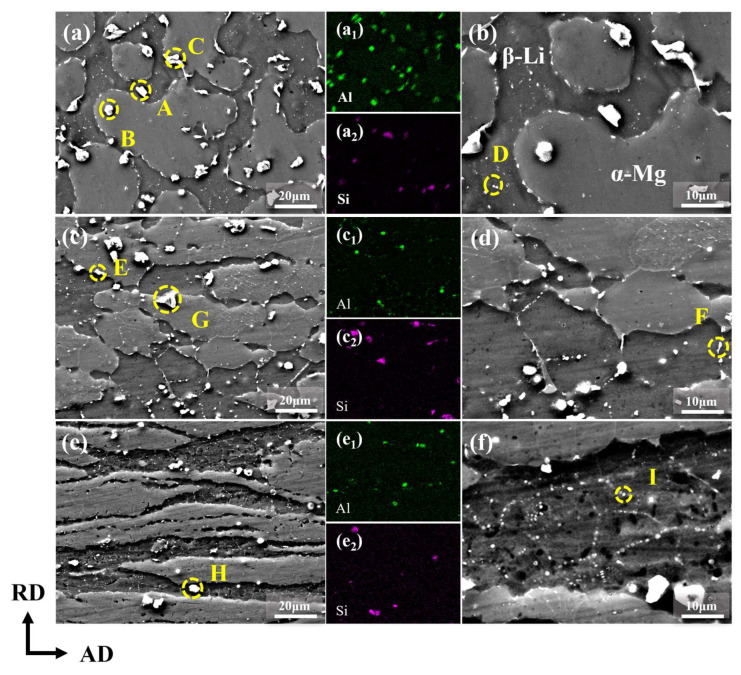
The SEM microstructure and EDS mapping of LAS830 alloy in as-cast and combination deformation: (**a**,**a_1_**,**a_2_**,**b**) as-cast; (**c**,**c_1_**,**c_2_**,**d**) BE; (**e**,**e_1_**,**e_2_**,**f**) SP.

Figure 5 illustrates the SEM images of the LAS830 alloy in as-cast and combination deformation. The as-cast alloy matrix is composed of bright gray α-Mg phase and dark gray β-Li phase, accompanied by a secondary phase of varying sizes and shapes, as shown in Figure 5a,b. In the EDS mapping images, Al is enriched within the β-Li phase and at the phase boundaries, forming spherical (point A) and strip-like (point C) phases. As displayed in Table 1, the proportion of Al atoms in point A and point C is close to 40%. Based on the XRD results from Figure 4 and existing studies, these are confirmed to be AlLi phases [12]. The irregular blocky phase (point B) within the α-Mg phase has a Mg-to-Si atomic ratio of approximately 2:1, which can be defined as the Mg_2_Si phase. Fine nanoscale phases (point D) distributed within the β-Li phase have an Al atomic percentage of about 10%. Based on the XRD and existing results, these are inferred to be MgLi_2_Al phases [24]. In the as-cast alloy, the AlLi phase is primarily distributed within the β-Li phase and at the α/β phase boundaries, while the Mg_2_Si phase is mainly found within the α-Mg phase and at the α/β phase boundaries, as shown in Figure 5a.

Figure 5c,d present the SEM microstructure of the LAS830 alloy after backward extrusion. The AlLi phase (point E) is refined and distributed in a punctate or strip-like pattern. Fine secondary phases (point F) appear at the grain boundaries within the β-Li phase, which, based on the EDS results in Table 1 and the XRD results, are identified as MgLi_2_Al phases. The α-Mg phase and the α/β phase boundaries still contain the Mg_2_Si phase (point G). After spinning, the AlLi phase is further refined, the grains within the β-Li phase become even finer, and more MgLi_2_Al phases (point I) appear in the grain boundaries. Additionally, the Mg_2_Si phase (point H) exists on the β-Li phase, and EDS point scanning reveals an enrichment of Al elements in this Mg_2_Si phase.

**Figure 6:**
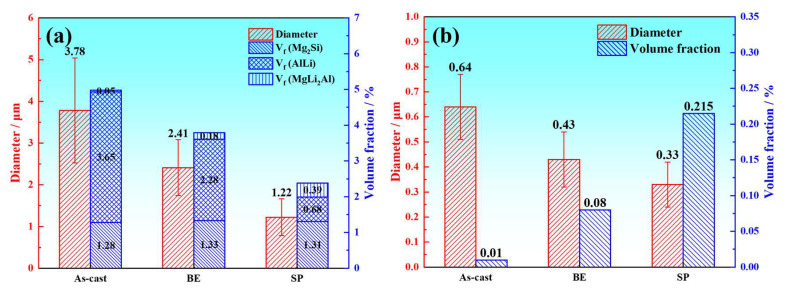
The second phase size and volume percent of LAS830 alloy in as-cast and combination deformation: (**a**) the matrix alloy as a whole; (**b**) inside the β-Li phase.

It is evident from the above that the combined deformation, which includes backward extrusion and spinning, results in the refinement of the alloy’s secondary phases. However, as can be known from Figure 5d,f, the fine MgLi_2_Al phase is primarily located at the grain boundaries within the β-Li phase. Therefore, this work statistically analyzed the size and volume percent of the matrix second phase and the second phase within the β-Li phase, with the results shown in Figure 6. The results indicate that the average size of the second phase in the as-cast alloy is 3.78 μm, and the volume percent is 4.95%, both being the highest. With increasing deformation, the second phase shows a decrease in the mean size and volume percent. Since the Mg_2_Si phase is a hard thermal stable phase, its volume fraction does not change much. After spinning, the mean size of the secondary phase is determined to be 1.22 μm, with a volume percent of 2.38%. This suggests that the second phase is refined under the action of combined deformation. And from Figure 6a, it can be seen that the second phase is mainly the refinement of the AlLi phase. Concurrently, the mean size of the second phase within the β-Li phase gradually decreases with increasing deformation, from 0.64 μm in the as-cast alloy to 0.33 μm after spinning. However, its volume percent increases, from 0.01% to 0.215%. This indicates that combined deformation can promote the precipitation of MgLi_2_Al phase.

**Figure 7:**
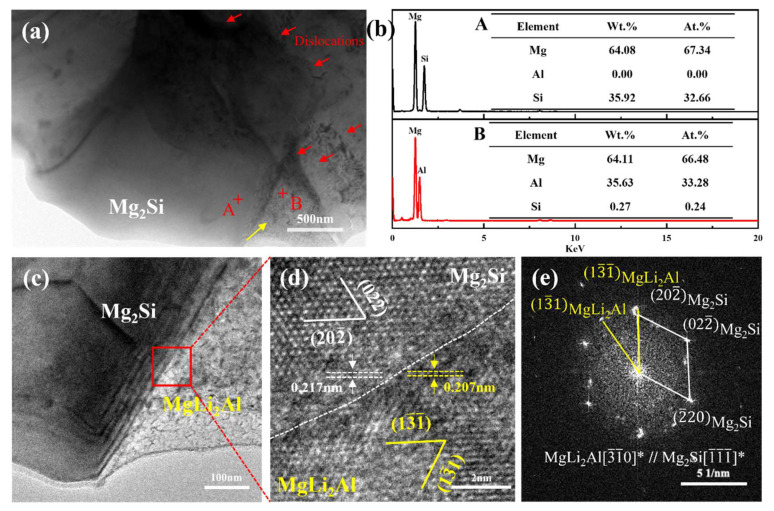
The TEM microstructure of the LAS830 alloy after spinning: (**a**) bright field image; (**b**) EDS results (at%) of point A and point B in (**a**); (**c**) bright field image; (**d**) HRTEM image from the red area in (**c**); (**e**) the corresponding SAED pattern of (**d**). "*" represents the crystal band axis calibrated by the two-phase diffraction spots.

It can be known that after spinning, the Mg_2_Si phase is found within the β-Li phase from Figure 5(e,e_1_,e_2_), and there is local concentration of Al elements, which indicates that Al-rich phases precipitate in the nearby region. To determine the phase composition, a TEM microstructural analysis was conducted on the SP alloy, as shown in Figure 7a. Point scanning was performed on the black blocky phase, and the EDS results are depicted in Figure 7b, with a ratio of atoms in Mg to Si is close to 2:1, confirming it as the Mg_2_Si phase. In Figure 7a, high-density dislocations are observed around the Mg_2_Si phase, with a large blocky phase present on the right side (indicated by the yellow arrow). The EDS result of point B on the right phase revealed an atomic percentage of Al to be 33.28%. Figure 7d presents the high-resolution transmission electron microscopy (HRTEM) image of the interface within the red area shown in Figure 7c. Subsequently, a Fourier transform was applied to the lattice image depicted in Figure 7d, resulting in the selected area electron diffraction (SAED) pattern at the interface, as illustrated in Figure 7e. It was determined from the SAED that both phases have a face-centered cubic (FCC) structure, with the interplanar spacing of ($1\overset{-}{3}1$) being 0.207 nm, close to the standard interplanar spacing of the MgLi_2_Al phase. The interplanar spacing for Mg_2_Si, specifically the ($20\overset{-}{2}$) plane, is 0.217 nm. Additionally, there are two crystallographic orientation relationships between the AlLi phase and the Mg_2_Si phase, namely ${(1\overset{-}{3}1)}_{{\mathrm{MgLi}}_{2}\mathrm{Al}}//{\left(20\overset{-}{2}\right)}_{{\mathrm{Mg}}_{2}\mathrm{Si}}$ and ${[\overset{-}{3}\overset{-}{1}0]}_{{\mathrm{MgLi}}_{2}\mathrm{Al}}//{\left[\overset{-}{1}\overset{-}{1}\overset{-}{1}\right]}_{{\mathrm{Mg}}_{2}\mathrm{Si}}$. The degree of mismatch between the two interfaces can be calculated using Equation (1) [25]:

(1) $$\delta =\frac{{d(20\overset{-}{2})}_{{\mathrm{Mg}}_{2}\mathrm{Si}}-{d(1\overset{-}{3}1)}_{{\mathrm{MgLi}}_{2}\mathrm{Al}}}{{d(20\overset{-}{2})}_{{\mathrm{Mg}}_{2}\mathrm{Si}}}\times 100\%$$

The value is approximately 4.61%, indicating a good atomic match between ${\left(20\overset{-}{2}\right)}_{{\mathrm{Mg}}_{2}\mathrm{Si}}$ and ${(1\overset{-}{3}1)}_{{\mathrm{MgLi}}_{2}\mathrm{Al}}$, with the interface relationship being coherent. This suggests that the MgLi_2_Al phase is capable of nucleating on the Mg_2_Si phase.

**Figure 8:**
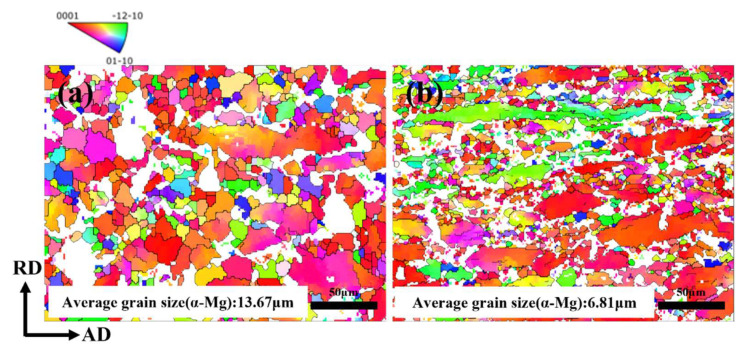
IPF diagrams of α-Mg phase in LAS830 alloy after backward extrusion and spinning: (**a**) BE; (**b**) SP.

In the above research work, it was observed that with the grains of the β-Li phase experienced an obvious refinement process as the composite deformation progressed. However, due to the limitations of the above technical means, the grain characteristics in the α-Mg phase cannot be effectively analyzed. In view of this, the α-Mg phase was investigated using the electron backscatter diffraction (EBSD) technique. Figure 8 shows the IPF diagram of α-Mg phase in BE alloy and SP alloy. Due to the active chemical properties of β-Li phase, it cannot produce diffraction patterns during EBSD test, so the white area is the influence area of zero-resolution β-Li phase and Mg_2_Si phase. In the BE alloy, the grain size of α-Mg is 13.67 μm, and after spinning, the grain size is refined to 6.81 μm. Moreover, it has also been observed that the α-Mg phase grains are elongated along the AD, which is due to the grains being inclined to deform along the AD when subjected to significant shear stress.

In summary, the β-Li phase is more easily deformed since there are more slip systems in β-Li (bcc) than α-Mg (hcp) [26]. After backward extrusion, high dislocation density and significant strain energy are produced in the β-Li phase, which is beneficial to the formation of recrystallized grains [26]. Furthermore, influenced by the dynamic recrystallization of β-Li phase, the deformation extent of the α-Mg phase is relatively minor, and its recrystallization process is consequently delayed. Furthermore, the energy of the new grain boundary is normally higher than the energy inside the grain boundary, resulting in faster migration and aggregation of atoms at the grain boundary, which promotes the precipitation of the MgLi_2_Al phase at the grain boundary [27]. Thus, during the β-Li recrystallization process, the MgLi_2_Al phase is precipitated at the grain boundary. Following the spinning process, the substantial deformation facilitates the refinement of the α-Mg phase grains. Concurrently, there is an increase in the precipitation of the MgLi_2_Al phase at the grain boundaries of the β-Li phase. Meanwhile, the precipitation of the MgLi_2_Al phase exerts a resisting effect on grain boundary migration, effectively restricting grain growth, thereby further refining the β-Li phase grains [28]. In addition, it is found that the MgLi_2_Al phase can also be precipitated by Mg_2_Si phase. Shi et al. [29] found that during the deformation process, particle deformation zones (PDZs) characterized by a high dislocation density and a significant orientation gradient forms in the vicinity of hard particles, which can promote the diffusion of atoms and facilitate the dynamic precipitation of the second phase. Since the Mg_2_Si phase is a thermally stable phase and is a hard particle, the deformation between the Mg_2_Si phase existing in the β-Li phase and the matrix is not coordinated, and it is prone to forming a high-density dislocation PDZ zone around the phase, which is instrumental in the diffusion of Al atoms and further promotes the formation of the MgLi_2_Al phase. In addition, Ma et al. [28] found that the second phase in the Mg-Li alloy is refined by element diffusion during deformation. Consistent with the results of this paper, with the progress of combination deformation, the second phase undergoes element diffusion, and the composite deformation leads to the increase in deformation time, so the mean size and volume percent of the second phase gradually decrease.

**Figure 9:**
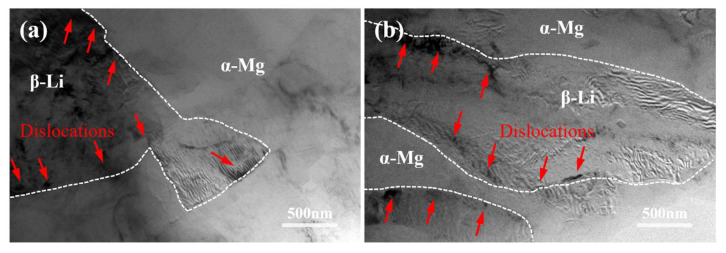
The phase boundary TEM microstructure of LAS830 alloy after backward extrusion and spinning: (**a**) BE alloy; (**b**) SP alloy.

Figure 9 shows the TEM images of the α/β phase boundaries in the LAS830 alloy after backward extrusion and spinning. A significant accumulation of dislocations at the α/β phase boundaries in the BE alloy can be observed. After spinning, the α/β phases are elongated, and the dislocation accumulation is distributed along the phase boundaries with a reduced density. Zhu et al.’s research [30] found that interfaces between hard and soft phases can impede dislocation motion. In this work, the hard α-Mg phase combines with the soft β-Li phase; thus, the α/β phase boundary can be considered as an interface between hard and soft phases that impedes dislocation motion.

### 3.2. Mechanical Properties

**Figure 10:**
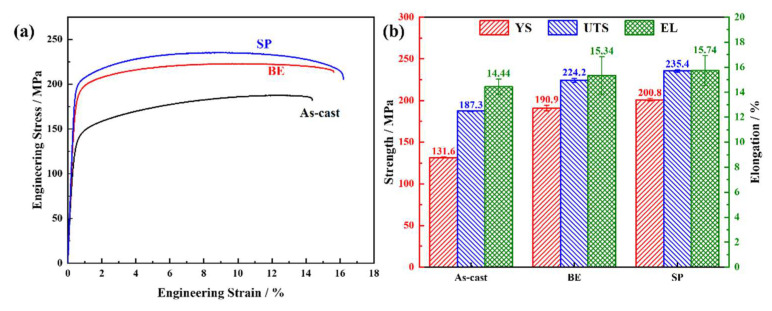
Mechanical properties of LAS830 alloy in as-cast and combination deformation: (**a**) engineering strain–stress curves; (**b**) bar chart of average YS, UTS, and EL value.

The room temperature tensile curves and corresponding data histograms of LAS830 alloy in as-cast and combination deformation are illustrated in Figure 10. The average yield strength (YS) and ultimate tensile strength (UTS) of the as-cast alloy are 131.6 MPa and 187.3 MPa, respectively, and the elongation (EL) is 14.44%. After backward extrusion, the strength and plasticity of the alloy are improved, and YS, UTS, and EL are increased to 190.9 MPa, 224.2 MPa, and 15.34%, respectively. After further spinning, both the strength and plasticity were further raised, and YS, UTS, and EL reached 200.8 MPa, 235.4 MPa, and 15.74%, respectively. The lamellar α/β structure is of benefit to the cooperative enhancement of plasticity and strength of the alloy [31], and the further refinement of the grains, along with the dispersion of the secondary phase, jointly contribute to the enhancement of both strength and plasticity [32].

**Figure 11:**
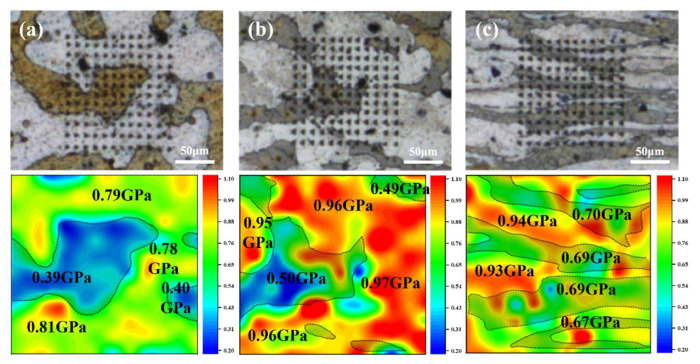
Cloud maps of nanoindentation hardness in LAS830 alloy at as-cast and combination deformation: (**a**) as-cast; (**b**) BE; (**c**) SP.

The nano-hardness test results of the LAS830 alloy in as-cast and combination deformation are shown in Figure 11. The dual-phase structure area is divided in the cloud diagram, in which the gray area is the β-Li phase, and the rest area is the α-Mg phase. In the three states, the average hardness of the α-Mg phase is higher than that of the β-Li phase. In as-cast alloy, the average hardness of the α-Mg phase and β-Li phase is 0.80 GPa and 0.40 GPa, respectively. After backward extrusion, the dual-phase structure is hardened, and the average hardness is increased to 0.98 GPa and 0.50 GPa, respectively. After spinning, the average hardness of the α-Mg phase decreases slightly, and the average hardness of the β-Li phase further increases to 0.69 GPa. With the progress of combination deformation, the volume percent of the secondary phase in the β-Li phase increases, which leads to precipitation strengthening.

### 3.3. Fracture Behavior

**Figure 12:**
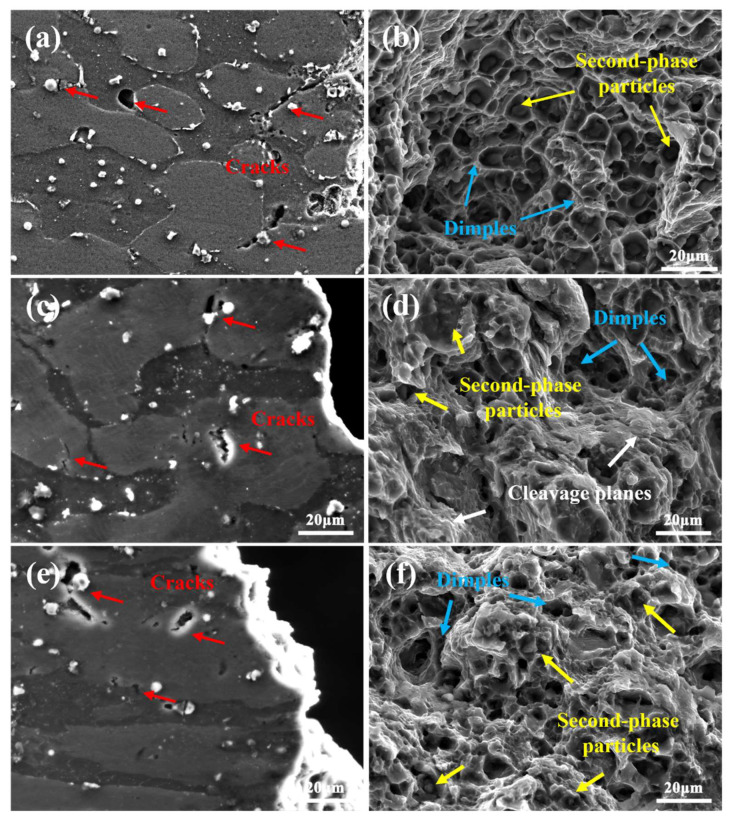
The side fracture morphology and tensile fracture surfaces of LAS830 alloy in as-cast and combination deformation: (**a**,**b**) As-cast; (**c**,**d**) BE; (**e**,**f**) SP.

The SEM microstructure of side fracture morphology and the tensile fracture surfaces of LAS830 alloy in as-cast and combination deformation are illustrated in Figure 12. From Figure 12a, it can be seen that during the tensile process of as-cast alloy, microcracks on the side fracture surface appear at the α-Mg and α/β phase boundaries, with the presence of the Mg_2_Si phase nearby (indicated by red arrows). Its tensile fracture surface (Figure 12b) contains many shallow and broad dimples (indicated by blue arrows), and one can observe secondary phase particles in the dimples (indicated by yellow arrows), indicating that ductile fracture was experienced by as-cast alloy during the tensile test. Compared to the as-cast alloy, fewer cracks at the phase boundaries and more cracks within the α-Mg phase are observed in the BE alloy, as shown in Figure 12c. In addition, the number and size of dimples decrease, and tearing ridges appear on the fracture surface (indicated by white arrows), exhibiting a ductile–brittle mixed fracture mode. After spinning, the cracks are mainly concentrated within the α-Mg phase, with a further increase in the number of cracks, which are smaller in size, as shown in Figure 12e. From the tensile fracture surface (Figure 12f), it can be seen that the size of dimples decreases and their number increases, with a small amount of secondary phase particles present at the base of the dimples., indicating ductile fracture.

The above phenomena indicate that during the tensile process, the Mg_2_Si phase is uncoordinated with the matrix deformation in the as-cast alloy, which is prone to stress concentration and results in the emergence of cracks [33]. When the crack propagates into the matrix, the stress concentration at the matrix is somewhat mitigated, resulting in minimal crack propagation. During the backward extrusion process, the β-Li phase, being more deformable than the α-Mg phase, leads to different deformation degrees between the two phases. Consequently, stress concentration is more likely to occur within the α-Mg phase during tension, causing crack initiation [34]. The significant hardness difference between the α-Mg and β-Li phases allows for the excellent coordination of deformation between the hard and soft phases. When the crack extends into the soft β-Li phase, it is effective to relieve the stress concentration by the plastic deformation of β-Li phase, slowing down and eventually stopping the crack propagation [34]. Therefore, micro-cracks in the BE alloy initiate in the α-Mg phase during the tensile test and subsequently arrest at the α/β phase boundaries. The lamellar structure facilitates stress redistribution via the interfaces between layers [35] in the SP alloy. Additionally, the hardness of the soft β-Li phase further increases, reducing the hardness difference between the α/β phases and impairing their coordinated deformation ability. Thus, during tension, stress concentration primarily occurs within the α-Mg phase [36], leading to crack initiation mainly occurring in the α-Mg phase, which delays crack initiation in the phase boundaries. Meanwhile, the stress concentration within the α-Mg phase is not effectively relieved, so some cracks propagate into the β-Li phase without stopping and continue to grow or penetrate to form larger cracks within the β-Li phase.

As noted, after the LAS830 alloy experienced the combined deformation process of backward extrusion and spinning, the deformation degree of the α-Mg phase increased, transitioning from a reticular to a lamellar structure. The hardness difference between the α/β hard and soft phases decreases, weakening the β-Li phase’s ability to alleviate stress concentration. The crack initiation mode shifts from crack initiation at the second phase to crack initiation within the α-Mg phase itself, and the mitigation effect of the β-Li phase on crack propagation is diminished. Therefore, different microstructural morphologies and the hardness difference between the α/β hard and soft phases significantly influence the compatible deformation of the α/β dual-phase structure.

## 4. Discussion

Studies indicated that the plastic deformation of magnesium alloys is generally characterized by the proliferation of dislocations, leading to work hardening, as well as the annihilation of dislocations, resulting in softening [37]. To further explore the effects of combined deformation on the work hardening and softening behavior of LAS830 alloy, a thorough and in-depth discussion will be conducted in the subsequent sections.

### 4.1. Work Hardening Behavior

**Figure 13:**
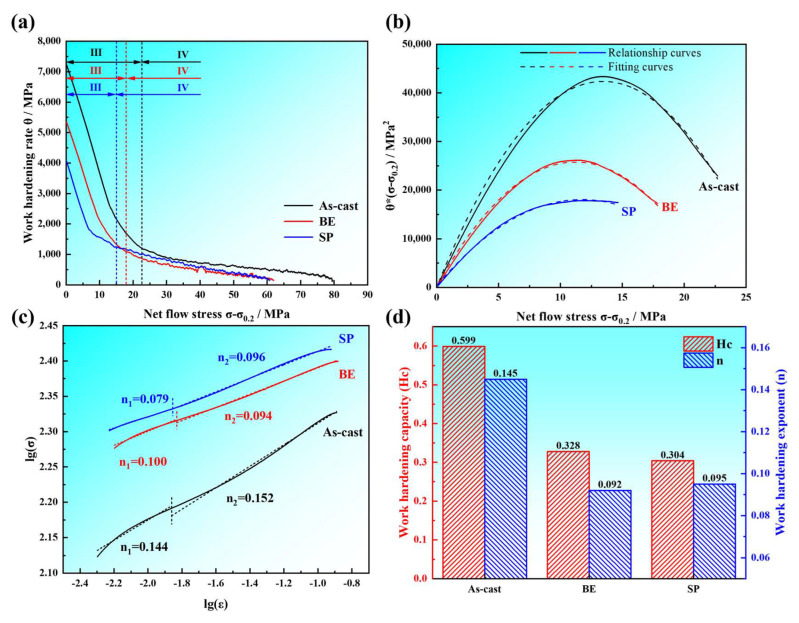
Work hardening curves of LAS830 alloy in as-cast and combination deformation: (**a**) *θ* and (*σ* − *σ*_0.2_) relationship curves; (**b**) *θ*(*σ* −*σ*_0.2_) and (*σ* − *σ*_0.2_) relationship curves; (**c**) true stress–strain logarithmic curves; (**d**) work hardening ability and the exponent.

Normally, the work hardening behavior of materials is typically characterized and quantified by examining the work hardening rate (*θ*) [38]:

(2) $$\theta =\frac{d\sigma }{d\varepsilon }$$

where *σ* denotes the true stress, and *ε* signifies the true strain. According to Equation (2), the variation curve of work hardening rate *θ* with flow stress (*σ* − *σ*_0.2_) can be drawn, as illustrated in Figure 13a. It was revealed that the alloy traverses two distinct phases of the work hardening behavior: a precipitous decline in the strain hardening rate, followed by a period of stable fluctuation in the strain hardening rate. These correspond, respectively, to the dynamic recovery stage (Stage III) and the large strain hardening stage (Stage IV) in the Kocks–Mecking model [39]. The dotted line position in Figure 13a is the corresponding stage turning point. In Stage III, with the progressive escalation of the flow stress, the work hardening rate of the alloy exhibits a linear diminution due to the activation of dislocation cross slip and the occurrence of dynamic recovery [40]. From Figure 13a, it is evident that the as-cast alloy possesses the utmost work hardening rate, which gradually decreases with increasing deformation degree. The flow stress corresponding to the stage turning point between Stage III and Stage IV is also gradually decreasing. Furthermore, in Stage IV, both the work hardening rate and the rate of its decrease for the SP alloy are higher in comparison to those observed for BE alloy.

Generally, the work hardening behavior during plastic deformation is closely related to dislocation density, such that the work hardening rate rises as the dislocation density increases. [41]. Cao et al. [42] demonstrated that the decrease in the hardness of the hard phase distributed in a lamellar structure leads to interfaces that tend to absorb dislocations and release stress. Similar results are observed in this work, as shown in Figure 9, where dislocation pile-ups are present at α/β phase boundaries, with fewer pile-ups in the SP alloy. Additionally, Li et al. [43] showed that after rolling, the microstructure of the Mg-8Li-3Al-0.3Si alloy exhibits lamellar distribution. In comparison with the as-cast alloy, there is a reduction in the work hardening rate. Moreover, the work hardening rate subsequent to rolling alloys diminishes as the layer spacing is reduced. As described in Section 3.1, the combined deformation gradually transforms the dual-phase structure of the alloy from a reticular distribution to lamellar distribution, leading to a decrease in the work hardening rate. Furthermore, in contrast to the BE alloy, the SP alloy exhibits an augmented aspect ratio of the α-Mg phase, reduced interlayer distance, slightly lower average hardness, and less dislocation accumulation at α/β phase boundaries, which alleviates stress concentration and further reduces the rate of work hardening.

Furthermore, the work hardening rate is significantly influenced by grain size and the presence of second phases [38]. Li et al. [44] found that the work hardening rate is decreased as a consequence of the depletion in grain size of Mg alloys. During tensile deformation, coarse grains are capable of accommodating a substantial quantity of newly generated dislocations, while the dislocation density in fine grains easily reaches a saturated state, thereby reducing the work hardening rate. In this work, the grain size of the dual-phase microstructure gradually decreases during the combined deformation process, resulting in a reduced rate of work hardening. Moreover, studies showed that the work hardening rate is effectively increased by the pinning effect of second phases on dislocations [45]. From Figure 6a, it can be observed that the volume percent of second phases gradually decreases with increasing deformation, this is not conducive to the pinning effect on dislocations. Consequently, the work hardening rate diminishes as a result of the reduction in second phase size and the concurrent decrease in volume percent. Thus, due to the synergistic effects of the α/β lamellar structure’s coordinated deformation and grain refinement, the work hardening rate of the LAS830 alloy declines as deformation increases.

The model established by Lukac et al. [39] can analyze the change in dislocation density *ρ* with strain *γ*, which can be used to further analyze the effect of composite deformation on the dynamic recovery stage of LAS830 alloy, with the mathematical expression being as follows:

(3) $$\frac{d\rho }{d\gamma }=k+k_{1}\rho^{1/2}-k_{2}\rho -k_{3}\rho^{2}$$

In this equation, *k =* (*bd*)^−1^, *b* represents the Burgers vector and *d* denotes the distance of non-dislocation obstacles that contribute to enhancing the moving resistance of dislocations. The parameter *k*_1_ is intricately linked to the interactions among dislocations, whereas *k*_2_ is related to dynamic recovery due to dislocation cross-slip, and *k*_3_ is associated with dynamic recovery resulting from dislocation climb. The correlation between *dρ*/*dγ* and *ρ*^1/2^ can be represented by the interrelation between *θ*(*σ* − *σ*_0.2_) and (*σ* − *σ*_0.2_); hence, Equation (3) can also be expressed as follows:

(4) $$\theta \left(\sigma -\sigma_{0.2}\right)=k+k_{1}\left(\sigma -\sigma_{0.2}\right)-k_{2}{\left(\sigma -\sigma_{0.2}\right)}^{2}-k_{3}{\left(\sigma -\sigma_{0.2}\right)}^{4}$$

Activating dislocation climb in magnesium alloys during tensile deformation at room temperature proves to be difficult, hence *k*_3_ = 0. The relationship curves between *θ*(*σ* − *σ*_0.2_) and (*σ* − *σ*_0.2_) is shown in Figure 13b. As plastic deformation progresses, each condition obviously sees the dislocation density of the alloy first increase and then decrease. By fitting Figure 13b according to Equation (3), the values of the parameters are obtained, and Table 2 shows the numerical results.

From Table 2, it can be observed that with the progress of combination deformation, the *k* value is positive correlation trend, and the *k_1_* and *k_2_* values are negative correlation trend. This occurs because as the progress of combination deformation, the size of the second phase particles within the alloy decreases, and the volume percent of MgLi_2_Al phase within the β-Li phase increases, leading to an increased obstruction to dislocation motion during Stage III in the tensile process. Meanwhile, the interaction between dislocations is weakened, and dislocation cross-slip causes a reduction in recovery efficiency.

Typically, the work hardening capacity (*Hc*) serves as a representation of the degree of work hardening in metallic materials [46,47], which can be calculated using the following equation:

(5) $$Hc=\frac{\sigma_{UTS}-\sigma_{0.2}}{\sigma_{0.2}}$$

where *σ_UTS_* represents the true ultimate tensile strength and *σ*_0.2_ represents the true yield strength, respectively. The corresponding numerical results are depicted in Figure 13d. The results indicate that as the degree of deformation increases, the *Hc* gradually decreases. N. Afrin et al. [48], based on the Hall–Petch formula, suggested that the reduction in grain size leads to a smaller disparity in flow stress between the grain boundaries and the interior of the grains, thereby leading to a decrease in hardening capacity. Additionally, in the study by Cao et al. [42], it was found that the lamellar distribution of hard phases and the reduction in their hardness can alleviate stress concentration at the interfaces, resulting in a reduction in *Hc*. To sum up, as the degree of deformation increases, the α-Mg phase progressively transitions to a lamellar structure, and the grain size gradually decreases, leading to a gradual reduction in *Hc*.

Moreover, the work hardening exponent (*n*) can also effectively serve as a quantitative measure of the work hardening effect in the materials [49]:

(6) $$n=\frac{ln\sigma }{ln\varepsilon }$$

In the equation, *σ* and *ε* represent the true stress and true strain after yielding. By fitting the curves for Stage III, Stage IV, and the whole stage, Figure 13c,d illustrate the fitted curves along with the calculated values of *n*, respectively. The dotted line position in Figure 13c is the corresponding stage turning point. The results show that the work hardening exponent *n*_1_ is aligned with the trend of the work hardening capacity *Hc* during Stage III. Studies indicated [48] that the *n* value in Stage III and *Hc* value are influenced by the same factors, as both exhibit a decline as the grain size is reduced. Therefore, in this work, as the progress of combination deformation and grain size gradually decreases, the work hardening exponent *n*_1_ also gradually decreases. In Stage IV, the *n*_2_ value of BE alloy is 0.094, and it slightly increases after spinning. Similar to the results in Figure 13a, when the alloy enters Stage IV, in contrast to BE alloy, the SP alloy demonstrates a higher work hardening rate and a more rapid rate of decrease. The varying rates of decrease in the work hardening rate could potentially be associated with the presence of internal precipitates and the grain size [40]. The strong absorption effect of grain boundaries on dislocations likely contributes to the reduction in the work hardening rate. The SP alloy has smaller grain sizes than the BE alloy, with a stronger ability to absorb dislocations, resulting in a higher rate of decrease in work hardening rate. It is worth mentioning that fine precipitates can hinder the migration of dislocations or grain boundaries, thereby increasing the work hardening rate [50]. In summary, in Stage IV, the smaller grains in the SP alloy lead to lower dislocation accumulation, and the smaller grains have a stronger ability to absorb dislocations, resulting in a better rate of decrease in work hardening rate. As the tensile process proceeds, the impact of grain boundaries on the work hardening rate can be offset by the fine and dispersed nanophase. Thus, the disparity in the rate of decrease in the work hardening rate between the two alloys can be attributed to the differences in precipitates and grain size. Similarly, in Stage IV, the fine and dispersed nanophase in the SP alloy hinders the motion of dislocations, leading to an elevation in dislocation density, which, in turn, amplifies the work hardening exponent. Moreover, the change trend in the n value in the whole stage of the material is consistent with that in the fourth stage, which is reduced after backward extrusion and increased after spinning. It can be seen that the overall work hardening exponent of the alloy is mainly dominated by the deformation of the fourth stage, which is affected by the fine dispersed phase, resulting in the increase in *n* value in the SP alloy.

### 4.2. Work Softening Behavior

**Figure 14:**
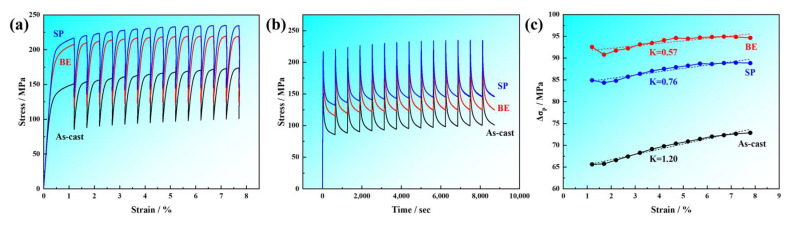
Relaxation softening behavior of LAS830 alloy in as-cast and combination deformation: (**a**) curve for the cyclic stress over strain; (**b**) curve for the cyclic stress over time; (**c**) variational curve for Δ*σ_p_* with strain.

The curves for the cyclic stress over the strain of the LAS830 alloy after cyclic stress relaxation testing are presented in Figure 14a. It can be observed from the curves that when the strain is maintained at a constant level, the stress experiences a rapid decline, indicating the presence of a softening effect. The decrease in stress observed during each cycle of the stress relaxation test results from the annihilation or rearrangement of dislocations, leading to a reduction in dislocation density [51]. In addition, in the SP alloy, due to the influence of the lamellar soft and hard α/β phase interface and fine grains, the dislocation motion is blocked during the cyclic stress relaxation process, thereby improving the cyclic stress–strain curve level of SP alloy. Figure 14b presents the stress–time curves after cyclic stress relaxation testing, from which it can be seen that with the increase in relaxation time, the softening rate progressively reduces. The stress drop value Δ*σ_p_*, is a key parameter for measuring the softening behavior and can be used to analyze the differences in softening behavior of the alloy under different conditions [37]. The calculation formula is as follows:

(7) $${∆\sigma }_{p}=\sigma_{0}-\sigma_{t}$$

Here, *σ*_0_ and *σ_t_* are the start stress and end stress of each cycle, respectively. Figure 14c illustrates the variation curves of stress drop Δ*σ_p_* per cycle, as a function of strain. It was revealed that the smallest stress drop Δ*σ_p_* is exhibited by the as-cast alloy, while BE alloy shows the highest, and SP alloy shows a decrease. This implies that the deformed alloys are more susceptible to softening.

The softening behavior of magnesium alloys, like work hardening behavior, is influenced by factors such as second phase particles, grain size, and dislocation slip mechanisms [43]. Research by Shi et al. [45] showed that grain boundaries in fine grains more readily absorb dislocations, thereby enhancing the material’s softening efficiency. The time (*τ*) expression of the grain boundaries absorbing dislocation is as follows:

(8) $$\tau =\frac{\rho bd}{\alpha \dot{\varepsilon }}$$

In the equation, *ρ* represents the dislocation density, $\dot{\varepsilon }$ is the strain rate, *α* and *b* are a constant and the Burgers vector, respectively. From Equation (8), it can be seen that the time of absorbing dislocations is shortened with the reduction in grain size for grain boundaries, which promotes softening. In this work, with the progress of combination deformation, its grain size decreases accordingly, thereby reducing the time of absorbing dislocations for grain boundaries, and thus the alloy exhibits a greater degree of softening after deformation compared to the as-cast state. However, research by Hou et al. [38] found that fine second phases can hinder dislocation cross-slip and reduce dislocation recovery, thereby reducing the softening effect. The SP alloy contains more precipitates within the β-Li phase than the BE alloy, which exerts a stronger obstructive effect on dislocation motion; hence, the degree of softening in the SP alloy is lower than that in the BE alloy. The SP alloy has more nanoscale precipitates than the BE alloy, which hinders dislocation motion, thereby diminishing the promoting effect of fine grain boundaries on the softening process of the alloy. Furthermore, Liu et al. [52] demonstrated that the soft/hard phase lamellar structure allows stress to be more effectively redistributed through layer interfaces during deformation, reducing the energy stored within the composite plate and consequently decreasing the degree of softening. It is known that the SP alloy with α/β lamellar distribution stores less energy, leading to a reduced degree of softening. Therefore, the combined deformation reduces grain size, resulting in a greater degree of softening in the deformed alloy, but due to the influence of fine dispersed second phases and α/β lamellar distribution, the degree of softening in SP alloy is lower than that in BE alloy.

From Figure 14c, it can be observed that as the strain increases, the overall Δ*σ_p_* of the alloy shows a rising trend. The high stored energy introduced by work hardening during the tensile process can provide softening behavior with an effective driving force [53]; hence, as the strain increases, Δ*σ_p_* rises. However, the upward trend varies among different conditions. The change rate of Δ*σ_p_* can be expressed using Equation (9):

(9) $$K=\frac{\partial \left({∆\sigma }_{p}\right)}{\partial \varepsilon }$$

Figure 14c illustrates the calculation results. For the as-cast alloy, the *K* value is at its peak, measuring 1.20. In contrast, the BE alloy has a reduced *K* value of 0.57, and after spinning, the *K* value increases to 0.76. Zhang et al. [54] found that the lamellar distribution of hard phases can alleviate stress concentration and contribute to softening behavior. Additionally, the work hardening effect formed during plastic deformation can provide more substantial impetus for the promotional effect of softening behavior [38]. As shown in Figure 13a, the work hardening rate of the as-cast alloy is the highest in Stage IV, thus providing the greatest driving force for softening and, consequently, the highest *K* value. The SP alloy has a work hardening rate that is better than that of the BE alloy in Stage IV. Therefore, the impetus induced by work hardening is better in the SP alloy than in the BE alloy, and the lamellar distribution further promotes softening, leading to a higher rate of softening increase.

## 5. Conclusions

This work prepared the LAS830 alloy using the vacuum melting method and investigated the effects of combined deformation (backward extrusion and spinning) on the microstructure and mechanical properties of the LAS830 alloy, as well as the impact of combined deformation on the work hardening and softening behavior of the alloy. The primary conclusions drawn are as follows:

(1) The deformation from backward extrusion enables the LAS830 alloy to exhibit a reduction in grain size; refinement, with a decreased volume percent of the AlLi phase; and the precipitation of the MgLi_2_Al phase within the β-Li phase. After spinning, the α/β phases are distributed in layers, with further grain refinement and increased precipitation of the MgLi_2_Al phase.
(2) The YS, UTS, and EL of the LAS830 alloy increased with the progress of combination deformation, reaching the ultimate values of 200.8 MPa, 235.4 MPa, and 15.74%, respectively. The higher hardness difference between the α/β phases after backward extrusion is beneficial for alleviating stress concentration. The lamellar distribution of the α/β phases and the reduction in hardness difference induced by spinning lead to crack initiation primarily within the α-Mg phase.
(3) The work hardening rate of the deformed LAS830 alloys was reduced. The spinning process decreases the work hardening rate during the dynamic recovery stage compared with the alloy after backward extrusion, due to the α/β lamellar structure, while the increased number of nanoscale precipitates enhanced the work hardening rate during the large strain hardening stage.
(4) The degree of softening during stress relaxation in the combination deformation alloy increased. Spinning, due to the α/β lamellar structure and finer grains, resulted in a lower softening rate compared to the backward extruded alloy, but the softening growth rate was greater than that observed in the backward extruded alloy, influenced by higher work hardening rate and α/β lamellar distribution.

## Figures and Tables

**Table 1:** The EDS results (at%) of the yellow-labeled second phase in Figure 5.

Positions	Mg (at. %)	Al (at. %)	Si (at. %)
A	55.8	44.1	0.1
B	69.0	0.8	30.2
C	60.7	39.8	0.5
D	89.1	10.7	0.2
E	57.9	42.1	0.0
F	74.3	25.6	0.0
G	62.6	25.0	12.4
H	48.2	27.2	24.5
I	90.2	9.8	0.0

**Table 2:** Fitting parameters for strain hardening *θ*(*σ* − *σ*_0.2_) and (*σ* − *σ*_0.2_) of LAS830 alloy in as-cast and combination deformation.

States	k	k_1_	k_2_	R^2^
As-cast	100 ± 32	6289 ± 13	234 ± 0.7	0.999
BE	280 ± 45	4552 ± 07	203 ± 0.5	0.999
SP	350 ± 42	3022 ± 12	129 ± 0.7	0.998

## Data Availability

The original contributions presented in this study are included in the article. Further inquiries can be directed to the corresponding author.
